# Correlation between Primary, Secondary Stability, Bone Density, Percentage of Vital Bone Formation and Implant Size

**DOI:** 10.3390/ijerph18136994

**Published:** 2021-06-30

**Authors:** Vasilena Ivanova, Ivan Chenchev, Stefan Zlatev, Eitan Mijiritsky

**Affiliations:** 1 Oral Surgery Department, Faculty of Dental Medicine, Medical University-Plovdiv, 4000 Plovdiv, Bulgaria; 2 Center of Dental Implantology, Research Institute at the Medical University of Plovdiv, 4000 Plovdiv, Bulgaria; ivan.chenchev@mu-plovdiv.bg; 3 CAD/CAM Center of Dental Medicine, Research Institute at the Medical University of Plovdiv, 4000 Plovdiv, Bulgaria; stefan.zlatev@mu-plovdiv.bg; 4 Head and Neck Maxillofacial Surgery, Department of Otoryngology, Tel-Aviv Sourasky Medical Center, Sackler Faculty of Medicine, Tel-Aviv University, Tel-Aviv 699350, Israel; mijiritsky@bezeqint.net

**Keywords:** dental implants, primary stability, secondary stability, bone density, implant diameter, implant length

## Abstract

Background: This study aims to evaluate whether there is a correlation between implant stability, bone density, vital bone formation and implant diameter and length. Methods: Ninety patients were enrolled in this study. They underwent a socket preservation procedure with allograft or PRF and after 4 months, a total of 90 implants were placed. CBCT scans were assigned prior to implant placement in order to assess the bone density. During the surgical re-entry, a bone biopsy was harvested with a trephine drill. Immediately after implant insertion, the primary stability was measured. The secondary stability was measured 4 months after implant placement. Results: Primary stability showed a significant positive linear correlation with bone density (r = 0.471, *p* < 0.001) as well as with percentage of new bone formation (r = 0.567, *p* < 0.001). An average significant association of secondary stability with bone density (rs = 0.498, *p* < 0.001) and percentage of newly formed bone (r = 0.477, *p* < 0.001) was revealed. The mean values of primary stability in all three implant sizes, regarding the diameter of the implants, were similar (narrow 67.75; standard 66.78; wide 71.21) with no significant difference (*p* = 0.262). The same tendency was observed for secondary stability (narrow 73.83; standard 75.25; wide 74.93), with no significant difference (*p* = 0.277). Conclusions: The study revealed a high correlation between primary and secondary implant stability, and bone density, as well as with the percentage of vital bone formation. Implant length and diameter revealed no linear correlation with the implant stability.

## 1. Introduction

One of the factors related to a successful dental implant treatment is the implant stability. It is determined by the quantity and the quality of bone tissue around the implant [1,2]. Primary stability is described as the result of bone compression during implantation. Good primary stability is essential for the prevention of implant micro-movements and in order for bone remodeling and osseointegration around the implant to be achieved [3]. Several studies have indicated that the tolerated micromotion threshold was found to be between 50 and 150 microns [4]. Davies et al. [5] found that excessive implant micromovements may affect the formation of a fibrin clot on the implant surface during the early healing process. Therefore, a good primary implant stability is a prerequisite for bone formation, osseointegration and increased bone–implant contact, which subsequently affects the secondary stability of the implant. [6] Secondary stability is referred to as biological stability that develops as a consequence of the formation and remodeling of alveolar bone on the implant surface [7,8].

There are a variety of methods that have been introduced to measure implant stability. Invasive methods include the assessment of removal torque (RT), and histological and histomorphometric studies. Although these methods provide objective data on the quality of bone implant contact, they are only used in an experimental setting [9]. Non-invasive methods include the percussion test, X-ray analysis, Periotest^®^ (Siemens AG, Bensheim, Germany), and resonance frequency analysis (RFA) [10]. Some of these methods are limited by their lack of standardization, poor sensitivity, and susceptibility to operator variables [11,12]. Resonance frequency analysis (RFA) has high clinical utility for monitoring the process of osseointegration due to its ease of use and the opportunity to perform multiple measurements [12].

In the present clinical study, the implant stability was measured with the Osstell Idx device (Gothenburg, Sweden). This RFA method is widely used in clinical practice for measuring implant stability and providing useful information in cases involving early loading or immediate implant placement [13,14].

Another important factor which determines successful osseointegration is the bone density. Schwartz et al. [15] introduced the concept of using computed tomography (CT) for the preoperative evaluation of alveolar bone density in patients undergoing implant placement. Compared to CT, CBCT has various advantages, including high resolution, lower radiation dose and reduced costs. Nevertheless, this method has some disadvantages, such as scattered radiation, the limited dynamic range of X-ray detectors, and different calibrations [16]. There is a strong correlation between the gray values of CBCT and Hounsfield units of multilayer CT [17,18,19]. Bone density influences primary stability; thus, the presence and thickness of the cortical bone may stabilize the implant because of its structural and mechanical properties [20,21].

Bone quality acting as one of the prerequisites for successful dental treatment, includes the physiological and structural aspects and the degree of bone mineralization [5]. In order to assess the internal bone structure, a histomoprhometric examination of bone biopsies is needed. It is performed with a trephine drill at the place of the dental implant. This procedure is considered as the “golden standard” method for the assessment of bone microstructure [22,23].

Finally, the impact of the implant size on the stability in different bone types should be taken into consideration. Degidi et al. [24] have suggested that the length and width of implants may affect primary stability due to the increased contact surface of the bone to the implant. Ostman et al. [25] reported a correlation between bone quality and primary stability, with lower ISQ values obtained for implants placed in softer bone. Lower ISQ values were observed when the length of the implants increased. There is no reported agreement on the impacts of the implant type or geometry as to how effectively they improve the primary stability in different bone types [26].

The aim of the present clinical study is to evaluate the correlation between primary, secondary stability and bone density, vital bone formation and implant size. The null hypothesis was that there is no correlation between the specified parameters. The null hypothesis was tested against the alternative hypothesis of significant correlation between them.

## 2. Materials and Methods

### 2.1. Study Design

The present clinical study was performed according to the ethical principles outlined in the Declaration of Helsinki. The ethical committee of the Medical University of Plovdiv, Bulgaria approved the study protocol (ethic code: P-2230/26.04.2018).

An a priori sample size calculation with pre-set *p*-value of 0.01 and power of 0.80 with a 3-group balanced design was performed using G*Power. The analysis determined a total of 90 required patients in order to satisfy the aforementioned parameters.

All of the participants underwent tooth extraction and socket preservation with different materials 4 months before implant placement. All implants (*n* = 90) were from 1 manufacturer (AB Dental Implants, Ashdod, Israel). In the upper jaw, 53 dental implants were placed, accounting for 58.90% of the entire sample, and in the mandible, 37 dental implants, which constituted 41.10% of the sample.

### 2.2. Radiographic Evaluation

Four months after tooth extraction and socket preservation procedure, a CBCT (Planmeca Romexis Viewer 4.4.3, Planmeca, Helsinki, Finland) was assigned. All CBCT assessments were performed using the same CBCT device. Repeated measurements were performed independently by two researchers in all three planes—axial, sagittal and coronal—and the mean HU values were determined.

### 2.3. Re-Entry Surgery and Implant Placement

**Figure 1:**
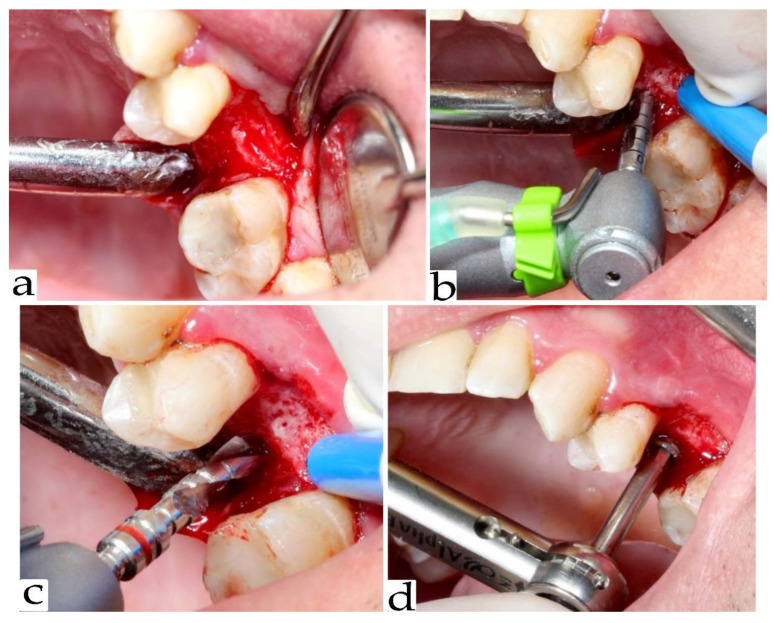
(**a**) Alveolar bone 4 months after tooth extraction and socket preservation (**b**) bone harvesting with trephine drill (**c**) implant osteotomy (**d**) insertion of the dental implant.

The healing time between the tooth extraction and socket preservation, and the implant placement procedure was 4 months. After this period, a surgical reentry was performed. After administering adequate local anesthesia, crestal and intrasulcular incisions were made. A mucoperiosteal flap was reflected to allow access to the alveolar bone. (Figure 1a).

A total of 90 biopsies were harvested for histological evaluation. A trephine bur with a 2.5 mm internal diameter was used to take the core biopsies (Figure 1b). They were approximately 6 mm in length. Bone biopsies were fixed in a 10% neutral buffered formalin solution. Additional osteotomy was performed, and the dental implants were placed (Figure 1c,d).

### 2.4. Implant Stability

**Figure 2:**
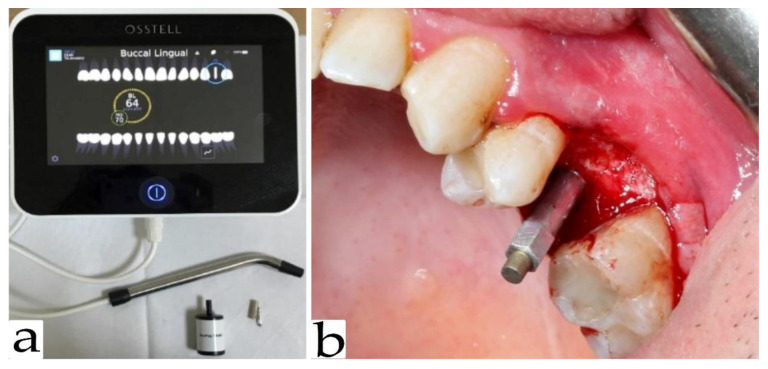
(**a**) Osstell Idx device (**b**) SmartPeg resonator attached to the implant.

Primary stability was measured immediately after implant placement in both mesiodistal and vestibulo-oral orientation using an Osstell Idx device (Figure 2a).

A SmartPeg resonator was attached to the implant (Figure 2b). The value obtained by the device was shown as an ISQ value. All values were saved and archived in the memory of the Osstell Idx. Four months after implant placement, the secondary implant stability was measured in a mesiodistal and bucco-oral direction using Osstell Idx apparatus. The measurements from both directions were calculated as a mean arithmetic between them.

### 2.5. Statistical Methods

For comparison of primary and secondary stability values between the three groups, the Kruskal–Wallis test was used, and for the post hoc pairwise comparisons, the Mann–Whitney U test. Descriptive statistics included median values, which, in the absence of a normal distribution, give a more reliable idea of the central tendency of primary and secondary stability values in the experimental groups.

The correlation between primary and secondary stability with bone density and the amount of vital bone formation was examined by correlation analysis. Primary stability values were correlated with bone density and vital bone formation via Pearson’s test. Regarding the secondary stability, correlation analysis was performed with the Spearman rank correlation test.

## 3. Results

### 3.1. Correlation between Primary and Secondary Stability with Bone Density and the Amount of Bone Formation in Biopsy Samples

Primary stability showed a significant positive linear correlation with bone density (r = 0.471, *p* < 0.001) according to Cohen’s reference limits (Cohen, 1988). A significant linear correlation was also found with the percentage of new bone formation, showing a high positive correlation (r = 0.567, *p* < 0.001).

**Figure 3:**
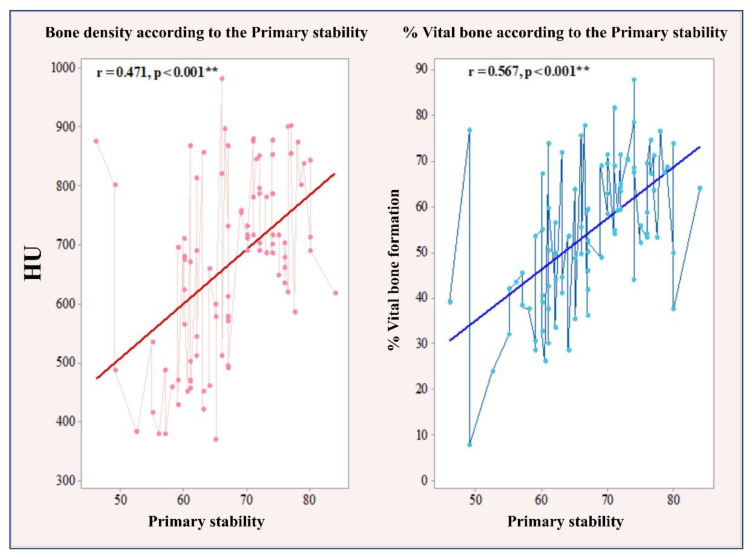
Correlation between primary stability and bone density and percentage of newly formed bone.

**Figure 4:**
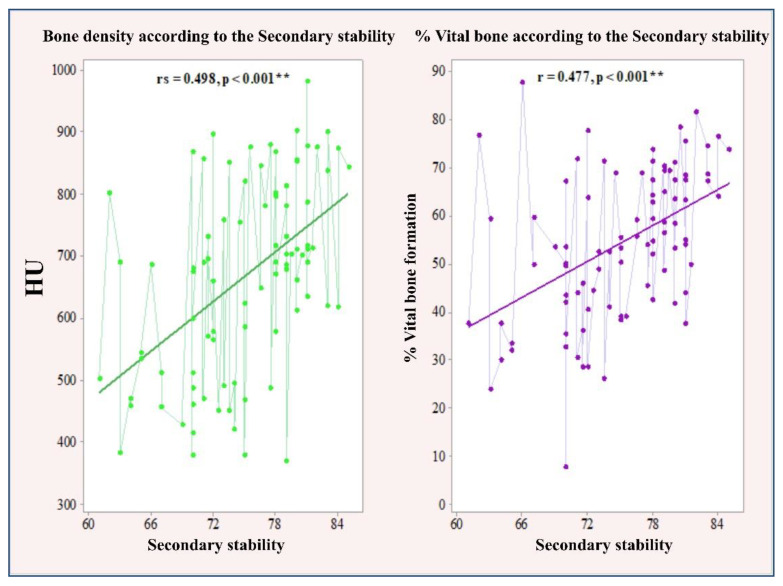
Correlation between secondary stability and bone density and percentage of newly formed bone.

The Pearson correlation test revealed an average significant association of secondary stability with bone density (rs = 0.498, *p* < 0.001) and percentage of newly formed bone (r = 0.477, *p* < 0.001). The significant linear correlation between primary and secondary stability and bone density and percentage of newly formed bone is illustrated in Figure 3 and Figure 4.

### 3.2. Correlation between Implant Size and Stability of the Implants

For the purpose of this analysis, the implants were distributed by diameter and length in the categories presented in Table 1. According to the diameter, 12 narrow implants (≤3.0 mm–<3.75 mm) were placed; 71 with standard size (≥3.75 mm–<5 mm) and 7 with wide size (≥5 mm). The percentage of standard-sized implants (79%) was significantly higher than the other two sizes (narrow 13%; wide 8%) according to the Fisher test, *p* < 0.001.

**Figure 5:**
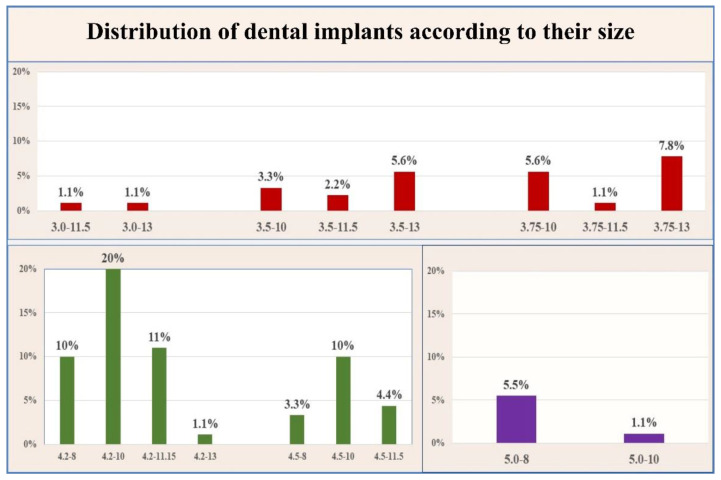
Distribution of the dental implants accordingt their size.

Depending on the length of the implant, they were distributed as follows: 17 short (≤6.0 mm), 59 standard (>6 mm–<10 mm) and 14 long (≥10 mm). Implants with standard size (65.50%) were prevalent in comparison to the other two groups (short 19%; long 15.50%), *p* < 0.001 (Figure 5).

**Figure 6:**
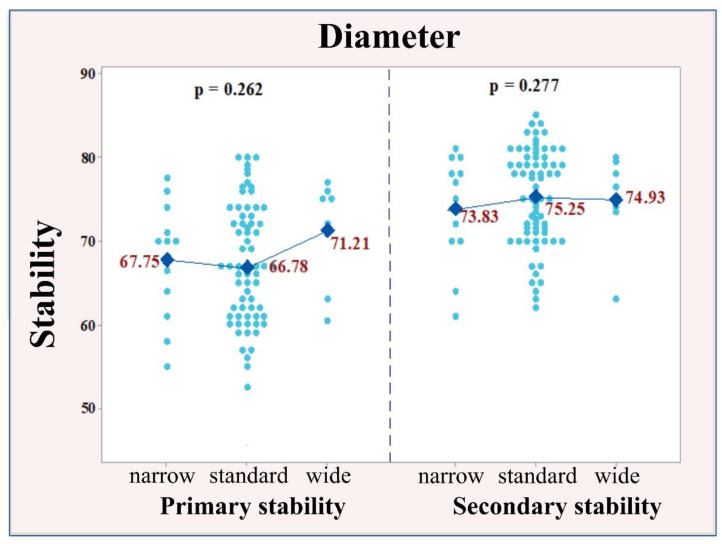
Distribution of the values of the primary and secondary stability according to the diameter of the implants.

А covariance analysis (ANCOVA) was performed to compare the mean values of primary and secondary stability according to the diameter (narrow, standard, wide) and implant length (short, standard, long). Тhe material used for socket preservation was considered as a covariate, which might affect primary and secondary stability. The mean values of primary stability in all three implant sizes, regarding the diameter of the implants, were similar (narrow 67.75; standard 66.78; wide 71.21) with no significant difference (*p* = 0.262). The same tendency was observed for secondary stability (narrow 73.83; standard 75.25; wide 74.93), with no significant difference, *p* = 0.277. The similar distribution of the individual values and the approximate mean values of primary and secondary stability according to the diameter of the implants are illustrated in Figure 6.

**Figure 7:**
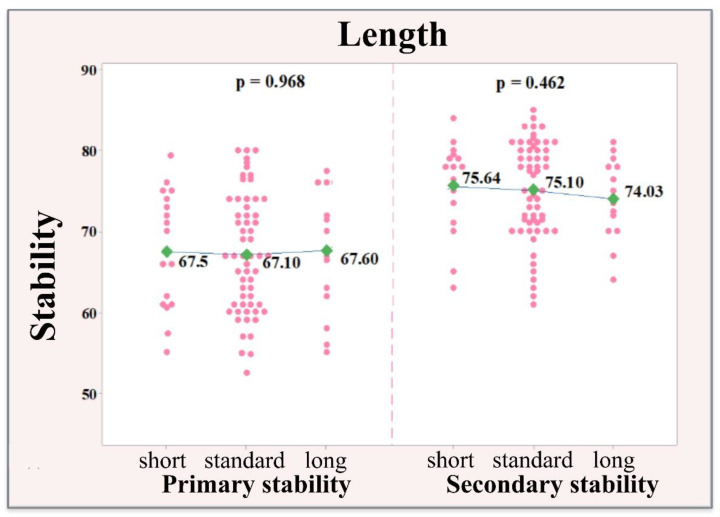
Distribution of the values of the primary and secondary stability according to the length of the implants.

No significant effect of implant length was found regarding the values of primary and secondary stability. With regard to primary stability, almost identical values are reported in terms of size (short 67.50; standard 67.10; wide 67.60), with no significant difference (*p* = 0.968). The secondary stability values are again very similar (short 75.64; standard 75.10; wide 74.04), with no significant difference (*p* = 0.462). The similarity in the values of primary and secondary stability when correlated to different implant sizes was valid regardless of the material used for socket preservation. The arithmetic mean values of primary and secondary stability according to the implant length are illustrated in Figure 7.

## 4. Discussion

A variety of factors influence the primary stability, including the bone quality and density at the placement site [27], the surgical technique [28,29], and the implant geometry, both micro- and macroscopic [4,30]. Successful dental implant treatment relies on both the quality and quantity of the available alveolar bone. [31]. The term ''bone quality'' has not been clearly defined in the literature. It includes physiological and structural aspects and the degree of bone mineralization [27]. The amount of newly formed bone and connective tissue can also affect different types of internal bone structure. A bone biopsy is required in order to evaluate these parameters. It was performed with a trephine bur at the place of the future implant. The first reported use of trephine bur for bone harvesting in implantology was in 1995 by Klinge et al. [32]. This procedure is reported as the “golden standard” among the methods for evaluating bone microstructure [33]. Performing a bone biopsy procedure is not carried out in daily practice due to the complexity of the manipulation, the prolonged operative time, and the presence of an insufficient volume of alveolar bone. In 1992, Sennerby et al. [34] postulated that the amount of cortical bone surrounding the implant was one of the most important factors for optimal implant stability. In the study of Rokn et al. [33] the authors reported that the tactile sensation of the surgeon may correlate with the histological properties of the bone; therefore, the practitioner is able to evaluate the healing prognosis of the bone at the time of implant placement. One of the aims of the present study was to determine whether there was a relationship between bone density and bone quality, represented as the amount of newly formed bone in the implant bed.

The extent of changes in bone quality is largely dependent on the duration of the healing process in addition to the resorption rate of the bone substitute and its ability to promote the formation of new bone [35]. The presence of residual graft material often interferes with the normal healing process and the clinical results [36]. The reduced bone density caused by the presence of residual particles can adversely affect the ability to obtain primary stability. This situation may occur when the remaining particles are encapsulated in the connective tissue [35]. Lekholm et al. [37] reported that 7.7% of the unsuccessful dental implant treatments were due to unsatisfactory structural characteristics of the alveolar bone. Trisi and Rao [22] were the first to attempt to find a correlation between bone density and histomorphometric analysis. They concluded that it is easy to recognize bone type D1 or D4, but it is difficult to differentiate between the two bone types—D2 and D3.

In a clinical study by Turkyilmaz et al. [38] the authors found a strong relationship between bone density and ISQ values. Herekar et al. [39] proposed an evaluation index according to which primary stability is 77.35 in bone D2, 70.55 in D3 bone and 60 in D4 bone. According to the research of Barikani et al. [26], the ISQ values of dental implants inserted in D1 bone are significantly higher than those of implants placed in D3 bone. According to Farré-Pagés et al. [40], there was a significant correlation between bone density and the ISQ values of the primary stability. Park et al. [41] performed an experimental animal model study, in which ISQ values had a significant correlation with the percentage of the bone–implant contact—BIC. Meanwhile, Rodrigo et al. [42] reported that the evaluation of RFA values (ISQ) had a statistically significant correlation with the success of implant placement. They did not report an implant with an ISQ > 60 that failed, while 19% of the implants with an ISQ < 60 failed. Pagliani et al. [43] proved that the correlation between implant micromotions and ISQ is not linear, and micromotions are reduced to about 50% at ISQ values between 60 and 70.

According to the results obtained in a study by Monje et al. [44], ISQ values changed throughout the osseointegration period after implant placement. Four months after implant placement, the average ISQ of 214 implants increased from 74.97 to 77.51. In a study by Da Cunha et al. [45], the mean values for the primary stability of 12 standard Brånemark system implants and 12 TiUnite Mk III Brånemark implants placed on the upper jaw were 67.9 ISQ. Ostman et al. [46] examined the primary stability of 906 dental implants. The average ISQ value they received for all implants was 67.4 ± 8.6. The authors found a correlation between alveolar bone quality and primary stability, with lower ISQ values obtained for implants placed in softer bone. The mean values obtained in their study are close to the ISQ values obtained in the present clinical study. Baftijari et al. [47] demonstrated a statistically significant increase in the secondary stability of 85 dental implants 3 months after placement. The results obtained by the authors revealed an ISQ of 58 (SD 5.91) for all implants placed in the anterior and posterior sectors of the upper jaw. The average ISQ value for secondary stability was 65 (SD 5.55). Rodrigo et al. [42], in their prospective clinical study of 4114 Straumann dental implants, evaluated the success of dental implants and concluded that there was no significant correlation between primary stability and implant success (*p* < 0.753) on the first day of surgery, but there was a significant difference (*p* < 0.001) by the day of recovery. The authors concluded that secondary stability is more important for the success of dental implants.

Previous research has shown that bone substitutes, or more precisely augmented bone, can provide good primary implant stability and that during surgery, only a few mechanical implant characteristics (length and diameter) are capable of influencing ISQ values [14]. Barikani et al. [26] reported that in D1 bone, the implant length did not make any significant difference in primary stability; however, in D3 bone, the primary stability of the implant increased when longer implants were utilized. Bilhan et al. [3] compared the effect of different implant diameters (mm) on the primary stability, and found no statistically significant differences in ISQ values. Carcia et al. [15] researched the effect of implant diameter on implant primary stability in D1 and D2 bone types and concluded that the implant diameter did not have any significant effects on primary implant stability.

These findings are in accordance with the clinical and histological observations of the present study. The present research demonstrated a significant correlation between the values of the primary stability, and the percentage of vital bone formation and bone density. The values of the correlation coefficients revealed an average significant association of secondary stability with bone density and the percentage of newly formed bone. Therefore, the null hypothesis of the correlation between the aforementioned parameters is approved. However, the implant size and diameter showed no significant effect regarding the values of primary and secondary stability.

## 5. Conclusions

The results of this clinical study indicated that primary and secondary implant stability is determined by bone density, as well as the percentage of vital bone formation. However, length and diameter offer no prediction value for the primary and secondary implant stability.

The clinical value of the current study includes the chances of using primary implant stability and bone density as factors for predicting the bone quality and the following successful implant treatment.

Further investigations regarding the parameters, which influence the long-term implant treatment, are necessary to determine the optimal treatment plan.

## Figures and Tables

**Table 1:** Distribution of the dental implants according to their length and diameter.

**Implant (I) Diameter**				**Fisher’s Test**
Term	Narrow	Standard	Wide	*p* < 0.001 **
Measurements	I < 3.75 mm	3.75 mm ≤ I ≤ 5 mm	I > 5	
Number (%)	12 (13%)	71 (79%)	7 (8%)	
**Implant (I) Length**				
Term	Short	Standard	Long	*p* < 0.001 **
Measurements	I ≤ 6 mm	6 mm < I < 10 mm	I ≥ 10 mm	
Number (%)	17 (19%)	59 (65.5%)	14 (15.50%)	

** Denotes significant difference.

## Data Availability

The data presented in this study are available on request from the corresponding author.
